# Supplementary material for: Interspecies Diversity of Osmotic Gradient Deformability of Red Blood Cells in Human and Seven Vertebrate Animal Species
Source: Cells. 2022 Apr 15;11(8):1351. doi: 10.3390/cells11081351 (PMC9026962; doi:10.3390/cells11081351)
Supplement: Supplementary file 1 [file cells-11-01351-s001.zip › cells-1666113-supplementary.pdf]

cells

MDPI

Supplementary Materials: Interspecies diversity of osmotic gradient deformability of red blood cells in human and seven vertebrate animal species

Adam Varga, Adam Attila Matrai, Barbara Barath, Adam Deak, Laszlo Horvath and Norbert Nemeth

Table S1. Summary of significantly differing hematological parameters in human, dog, cat, pig, sheep, mouse, rat and rabbit blood samples.

	**human**	**dog**	**cat**	**pig**	**sheep**	**mouse**	**rat**	**rabbit**
**human**	n.a.	WBC, RBC, Hgb, Hct, MCV, MCH, Plt: p<0.001	WBC: p=0.009, RBC, Hgb, Hct, MCV, MCH: p<0.001 MCHC: p=0.003	WBC, RBC, Hgb, Hct, MCV, MCH, MCHC, Plt: p<0.001	WBC, RBC, MCV, MCH, MCHC, Plt: p<0.001 Hgb: p=0.005	WBC, RBC, MCV, MCH, Plt: p<0.001 Hct: p=0.028	WBC, RBC, Hgb, Hct, MCV, MCH, Plt: p<0.001	WBC: p=0.035 Hct: p=0.002 RBC, Hgb, MCV, MCH, Plt: p<0.001
**dog**	WBC, RBC, Hgb, Hct, MCV, MCH, Plt: p<0.001	n.a.	RBC, Hgb, Hct, MCV, MCH, MCHC: p<0.001 Plt: p=0.004	RBC, Hgb, Hct, MCV, MCH, MCHC, Plt: p<0.001	WBC, RBC, Hgb, Hct, MCV, MCH, MCHC: p<0.001	WBC, RBC, Hgb, Hct, MCV, MCH, MCHC, Plt: p<0.001	WBC, RBC, Hgb, Hct, MCV, MCH, MCHC, Plt: p<0.001	WBC, RBC, Hgb, Hct, MCV, MCH, MCHC: p<0.001 Plt: p=0.019
**cat**	WBC: p=0.009 RBC, Hgb, Hct, MCV, MCH: p<0.001 MCHC: p=0.003	RBC, Hgb, Hct, MCV, MCH, MCHC: p<0.001 Plt: p=0.004	n.a.	RBC, Hgb, Hct, MCV, MCH, Plt: p<0.001	WBC, Hgb, Hct, MCV, MCH: p<0.001 RBC: p=0.004	WBC, RBC, Hgb, Hct, MCV, MCH, MCHC, Plt: p<0.001	WBC, RBC, Hgb, Hct, MCV, MCH, Plt: p<0.001 MCHC: p=0.004	WBC: p=0.002 RBC, Hgb, Hct, MCV, MCH, MCHC, Plt: p<0.001

**pig**	WBC, RBC, Hgb, Hct, MCV, MCH, MCHC, Plt: p<0.001	RBC, Hgb, Hct, MCV, MCH, MCHC, Plt: p<0.001	RBC, Hgb, Hct, MCV, MCH, Plt: p<0.001	n.a.	WBC, RBC, Hgb, Hct, MCV, MCH: p<0.001 Plt: p=0.033	WBC, RBC, Hgb, Hct, MCV, MCHC, Plt: p<0.001	WBC, Hgb, MCH, MCHC: p<0.001	WBC, Hgb, Hct, MCV, MCH, MCHC, Plt: p<0.001
**sheep**	WBC, RBC, MCV, MCH, MCHC, Plt: p<0.001 Hgb: p=0.005	WBC, RBC, Hgb, Hct, MCV, MCH, MCHC: p<0.001	WBC, Hgb, Hct, MCV, MCH: p<0.001 RBC: p=0.004	WBC, RBC, Hgb, Hct, MCV, MCH: p<0.001 Plt: p=0.033	n.a.	WBC, RBC, Hgb, MCV, MCH, MCHC, Plt: p<0.001	WBC, RBC, Hct, MCV, MCH, MCHC: p<0.001	WBC, RBC, MCV, MCH, MCHC: p<0.001 Hgb: p=0.03 Plt: p=0.044
**mouse**	WBC, RBC, MCV, MCH, Plt: p<0.001 Hct: p=0.028	WBC, RBC, Hgb, Hct, MCV, MCH, MCHC, Plt: p<0.001	WBC, RBC, Hgb, Hct, MCV, MCH, MCHC, Plt: p<0.001	WBC, RBC, Hgb, Hct, MCV, MCHC, Plt: p<0.001	WBC, RBC, Hgb, MCV, MCH, MCHC, Plt: p<0.001	n.a.	WBC, RBC, Hgb, Hct, MCV, MCH, Plt: p<0.001	RBC, Hct, MCV, MCH, Plt: p<0.001
**rat**	WBC, RBC, Hgb, Hct, MCV, MCH, Plt: p<0.001	WBC, RBC, Hgb, Hct, MCV, MCH, MCHC, Plt: p<0.001	WBC, RBC, Hgb, Hct, MCV, MCH, Plt: p<0.001 MCHC: p=0.004	WBC, Hgb, MCH, MCHC: p<0.001	WBC, RBC, Hct, MCV, MCH, MCHC: p<0.001	WBC, RBC, Hgb, Hct, MCV, MCH, Plt: p<0.001	n.a.	WBC, Hgb, MCV, MCH, Plt: p<0.001 Hct p=0.02
**rabbit**	WBC: p=0.035 Hct: p=0.002 RBC, Hgb, MCV, MCH, Plt: p<0.001	WBC, RBC, Hgb, Hct, MCV, MCH, MCHC: p<0.001 Plt: p=0.019	WBC: p=0.002 RBC, Hgb, Hct, MCV, MCH, MCHC, Plt: p<0.001	WBC, Hgb, Hct, MCV, MCH, MCHC, Plt: p<0.001	WBC, RBC, MCV, MCH, MCHC: p<0.001 Hgb: p=0.03 Plt: p=0.044	RBC, Hct, MCV, MCH, Plt: p<0.001	WBC, Hgb, MCV, MCH, Plt: p<0.001 Hct: p=0.02	n.a.

WBC: white blood cell count; Hct: hematocrit; RBC: red blood cell count; Hgb: hemoglobin; MCV: mean corpuscular volume; MCH: mean corpuscular hemoglobin; MCHC: mean corpuscular hemoglobin concentration, Plt: platelet count

Table S2. Summary of significantly differing conventional and osmotic gradient ektacytometry parameters in human, dog, cat, pig, sheep, mouse, rat and rabbit blood samples.

	**human**	**dog**	**cat**	**pig**	**sheep**	**mouse**	**rat**	**rabbit**
**human**	n.a.	EI at 3 Pa, EI_{max}, $SS_{1/2}$, $EI_{max}/SS_{1/2}$, EI min, O min, O (EI max), Area: p<0.001 EI max: p=0.003 EI hyper: p=0.005	EI at 3 Pa, EI_{max}, $SS_{1/2}$, EI_{max},/$SS_{1/2}$, O min, O (EI max), O hyper: p<0.001 Area: p=0.009	EI at 3 Pa, EI_{max}, $SS_{1/2}$, $EI_{max}/SS_{1/2}$, EI min, O (EI max), O hyper: p<0.001 O min: p=0.002 Area: p=0.005	EI at 3 Pa, EI_{max}, $SS_{1/2}$, $EI_{max}/SS_{1/2}$, EI max, EI hyper, O min, O (EI max), Area: p<0.001 O hyper: p=0.004	EI at 3 Pa, $SS_{1/2}$, $EI_{max}/SS_{1/2}$: p<0.001 O (EI max): p=0.003 O hyper: p=0.004	EI at 3 Pa, EI_{max}, $SS_{1/2}$, $EI_{max}/SS_{1/2}$, O hyper: p<0.001 EI min: p=0.004 EI max: p=0.0047 Area: p=0.015	EI at 3 Pa, EI_{max}, $SS_{1/2}$, $EI_{max}/SS_{1/2}$: p<0.001 EI min: p=0.004 EI max, EI hyper: p=0.012 O (EI max): p=0.034 Area: p=0.007
**dog**	EI at 3 Pa, EI_{max}, $SS_{1/2}$, $EI_{max}/SS_{1/2}$, EI min, O min, O (EI max), Area: p<0.001 EI max: p=0.003 EI hyper: p=0.005	n.a.	EI_{max}, $SS_{1/2}$, $EI_{max}/SS_{1/2}$, EI min, EI max, EI hyper, O min, O (EI max), O hyper, Area: p<0.001	EI_{max}, EI max, EI hyper, O min, O (EI max), O hyper, Area: p<0.001 $SS_{1/2}$: p=0.004	EI at 3 Pa, $SS_{1/2}$, EI_{max}, EI min, EI max, EI hyper, O min, O (EI max), Area: p<0.001 O hyper: p=0.01	EI_{max}, $SS_{1/2}$, EI min, O min, O (EI max), O hyper, Area: p<0.001 EI hyper: p=0.01 EI at 3 Pa: p=0.006	O min, O (EI max), O hyper: p<0.001 EI max: p=0.002 Area: p=0.012	$SS_{1/2}$, O min, O (EI max): p<0.001 EI at 3 Pa: p=0.011 $EI_{max}/SS_{1/2}$: p=0.031 EI min: p=0.043
**cat**	EI at 3 Pa, EI_{max}, $SS_{1/2}$, $EI_{max}/SS_{1/2}$, O min, O (EI max), O hyper: p<0.001 Area: p=0.009	EI_{max}, $SS_{1/2}$, $EI_{max}/SS_{1/2}$, EI min, EI max, EI hyper, O min, O (EI max), O hyper, Area: p<0.001	n.a.	$SS_{1/2}$, $EI_{max}/SS_{1/2}$, Area: p<0.001 EI min: p=0.003 O hyper: p=0.039	EI at 3 Pa, EI_{max}, $SS_{1/2}$, $EI_{max}/SS_{1/2}$, EI max, EI hyper, O min, Area: p<0.001 O (EI max):	EI at 3 Pa, EI_{max}, $EI_{max}/SS_{1/2}$, EI max, EI hyper, O min, O (EI max), Area:	EI_{max}, $EI_{max}/SS_{1/2}$, O (EI max), O hyper, Area: p<0.001 EI min: p=0.035	EI_{max}, $SS_{1/2}$, $EI_{max}/SS_{1/2}$, EI min, EI max, O min, O (EI max),

					p=0.011<br>EI min: p=0.014<br>O hyper: p=0.019	p<0.001<br>$SS_{1/2}$: p=0.043	O min: p=0.003<br>EI max: p=0.005<br>EI hyper: p=0.011<br>$SS_{1/2}$: p=0.043	Area: p<0.001<br>EI hyper: p=0.006<br>EI at 3 Pa: p=0.032
**pig**	EI at 3 Pa, EI_{max}, $SS_{1/2}$, $EI_{max}/SS_{1/2}$, EI min, O (EI max), O hyper: p<0.001<br>O min: p=0.002<br>Area: p=0.005	EI_{max}, EI max, EI hyper, O min, O (EI max), O hyper, Area: p<0.001<br>$SS_{1/2}$: p=0.004	$SS_{1/2}$, $EI_{max}/SS_{1/2}$, Area: p<0.001<br>EI min: p=0.003<br>O hyper: p=0.039	n.a.	EI at 3 Pa, $SS_{1/2}$, EI_{max}, EI max, EI hyper, O min, O (EI max), Area: p<0.001	EI at 3 Pa, $SS_{1/2}$, EI max, EI hyper: p<0.001<br>EI min: p=0.003<br>O (EI max): p=0.015	EI max, O min, O (EI max): p<0.001<br>$EI_{max}/SS_{1/2}$: p=0.009<br>Area: p=0.01<br>$SS_{1/2}$: p=0.024	EI_{max}, EI max, Area: p<0.001<br>O min: p=0.002<br>$EI_{max}/SS_{1/2}$, O (EI max): p=0.003<br>EI hyper: p=0.004<br>EI at 3 Pa: p=0.007<br>EI min: p=0.008
**sheep**	EI at 3 Pa, EI_{max}, $SS_{1/2}$, $EI_{max}/SS_{1/2}$, EI max, EI hyper, O min, O (EI max), Area: p<0.001<br>O hyper: p=0.004	EI at 3 Pa, $SS_{1/2}$, EI_{max}, EI min, EI max, EI hyper, O min, O (EI max), Area: p<0.001<br>O hyper: p=0.01	EI at 3 Pa, EI_{max}, $SS_{1/2}$, $EI_{max}/SS_{1/2}$, EI max, EI hyper, O min, Area: p<0.001<br>O (EI max): p=0.011<br>EI min: p=0.014<br>O hyper: p=0.019	EI at 3 Pa, $SS_{1/2}$, EI_{max}, EI max, EI hyper, O min, O (EI max), Area: p<0.001	n.a.	EI at 3 Pa, EI_{max}, $SS_{1/2}$, $EI_{max}/SS_{1/2}$, EI max, EI hyper, O min, O (EI max), Area: p<0.001<br>O hyper: p=0.003	EI at 3 Pa, $SS_{1/2}$, EI_{max}, EI min, EI max, EI hyper, O min, O (EI max), Area: p<0.001<br>O hyper: p=0.024	EI at 3 Pa, EI_{max}, $SS_{1/2}$, EI min, EI max, EI hyper, O min, O (EI max), Area: p<0.001<br>$EI_{max}/SS_{1/2}$: p=0.033
**mouse**	EI at 3 Pa, $SS_{1/2}$, $EI_{max}/SS_{1/2}$: p<0.001	EI_{max}, $SS_{1/2}$, $EI_{max}/SS_{1/2}$, EI min,	EI at 3 Pa, EI_{max}, $EI_{max}/SS_{1/2}$, EI max,	EI at 3 Pa, EI_{max}, $SS_{1/2}$, EI hyper:	EI at 3 Pa, EI_{max}, $SS_{1/2}$, $EI_{max}/SS_{1/2}$, EI	n.a.	EI_{max}, $SS_{1/2}$, $EI_{max}/SS_{1/2}$, O	EI min, EI max, O (EI max),

	O (EI max): p=0.003<br>O hyper: p=0.004	O min, O (EI max), O hyper, Area: p<0.001<br>EI hyper: p=0.01<br>EI at 3 Pa: p=0.006	EI hyper, O min, O (EI max), Area: p<0.001<br>$SS_{1/2}$: p=0.043	p<0.001<br>EI min: p=0.003<br>O (EI max): p=0.015	max, EI hyper, O min, O (EI max), Area: p<0.001<br>O hyper: p=0.003		hyper: p<0.001<br>EI at 3 Pa: p=0.004<br>O min: p=0.014<br>EI min: p=0.023	Area: p<0.001<br>O min: p=0.007<br>$SS_{1/2}$: p=0.009
**rat**	EI at 3 Pa, EI_{max}, $SS_{1/2}$, $EI_{max}/SS_{1/2}$, O hyper: p<0.001<br>EI min: p=0.004<br>EI max: p=0.0047<br>Area: p=0.015	O min, O (EI max), O hyper: p<0.001<br>EI max: p=0.002<br>Area: p=0.012	EI_{max}, $EI_{max}/SS_{1/2}$, O (EI max), O hyper, Area: p<0.001<br>O min: p=0.003<br>EI min: p=0.035<br>EI max: p=0.005<br>EI hyper: p=0.011<br>$SS_{1/2}$: p=0.043	EI max, O min, O (EI max): p<0.001<br>$EI_{max}/SS_{1/2}$: p=0.009<br>$SS_{1/2}$: p=0.024<br>Area: p=0.01	EI at 3 Pa, EI_{max}, $SS_{1/2}$, EI min, EI max, EI hyper, O min, O (EI max), Area: p<0.001<br>O hyper: p=0.024	EI_{max}, $SS_{1/2}$, $EI_{max}/SS_{1/2}$, O hyper: p<0.001<br>EI at 3 Pa: p=0.004<br>O min: p=0.014<br>EI min: p=0.023	n.a.	EI_{max}, $SS_{1/2}$: p<0.001<br>EI at 3 Pa: p=0.027
**rabbit**	EI at 3 Pa, EI_{max}, $SS_{1/2}$, $EI_{max}/SS_{1/2}$: p<0.001<br>EI min: p=0.004<br>Area: p=0.007<br>EI max, EI hyper: p=0.012<br>O (EI max): p=0.034	$SS_{1/2}$, O min, O (EI max): p<0.001<br>EI at 3 Pa: p=0.011<br>$EI_{max}/SS_{1/2}$: p=0.031<br>EI min: p=0.043	EI_{max}, $SS_{1/2}$, EI min, EI max, O min, O (EI max), Area: p<0.001<br>EI hyper: p=0.006<br>EI at 3 Pa: p=0.032	EI_{max}, EI max,<br>EI min: p=0.008<br>O min: p=0.002<br>O (EI max): p=0.003<br>EI hyper: p=0.004<br>Area: p<0.00<br>EI at 3 Pa: p=0.007<br>$EI_{max}/SS_{1/2}$: p=0.003	EI at 3 Pa, EI_{max}, $SS_{1/2}$, EI min, EI max, EI hyper, O min, O (EI max), Area: p<0.001<br>$EI_{max}/SS_{1/2}$: p=0.033	EI min, EI max, O (EI max), Area: p<0.001<br>O min: p=0.007<br>$SS_{1/2}$: p=0.009	EI_{max}, $SS_{1/2}$: p<0.001<br>EI at 3 Pa: p=0.027	n.a.

EI at 3 Pa: elongation index at 3 Pa, EI_{max}: calculated maximal elongation index, $SS_{1/2}$: shear stress at half EI_{max},
EI min: minimal elongation index in low osmolar environment, O min: osmolality at EI min, EI max: the maximal elongation index in the function of osmolality, O (EI max): the osmolality at EI max, EI hyper: half of the maximal elongation index at high osmolar environment, O hyper: osmolality at EI hyper, Area: calculated from the area under the individual elongation index-osmolality curves.
